# Polarization variable terahertz metasurface along the propagation path

**DOI:** 10.1016/j.fmre.2023.03.017

**Published:** 2023-04-28

**Authors:** Jitao Li, Jingyu Liu, Zhen Yue, Jie Li, Chenglong Zheng, Fan Yang, Hui Li, Yating Zhang, Yan Zhang, Jianquan Yao

**Affiliations:** a Key Laboratory of Opto-Electronics Information Technology (Tianjin University), Ministry of Education, School of Precision Instruments and Opto-Electronics Engineering, Tianjin University, Tianjin 300072, China; b Beijing Key Laboratory for Metamaterials and Devices, Key Laboratory of Terahertz Optoelectronics, Ministry of Education, and Beijing Advanced Innovation Center for Imaging Technology, Department of Physics, Capital Normal University, Beijing 100048, China

**Keywords:** Terahertz, Metasurface, Propagation path, Polarization manipulation, Longitudinal polarization, Refractive index sensing

## Abstract

Conventional metasurfaces for terahertz polarization are limited to performing lateral (in the *x-y* plane) polarization control of the output wave. In such cases, the polarization state remains unchanged in each output plane along the propagation path. Herein, we propose a terahertz polarization metasurface that operates longitudinally (i.e., along the *z*-axis direction of propagation), which modifies the polarization state of each output plane throughout the propagation path. Our designed metasurface can control the phase delays of the left-handed and right-handed circularly polarized (LCP and RCP) components of the incident terahertz wave. This enables the LCP beam and RCP beam to converge to the *z*-axis through distinct paths, creating a Bessel beam. The proposed design achieved a linearly polarized terahertz wave including both LCP and RCP components with a precise phase difference Δ*φ* at each point within a certain range along the *z*-axis. The Δ*φ* varies as the propagation distance, resulting in a rotated linearly polarized output wave along the propagation path, while the rotation angle ranges from 0 to π. Based on the variable property of longitudinal polarization, we propose an application concept of dielectric refractive index sensing, in which an additional medium is placed in the terahertz propagation path and the unknown refractive index is determined by detecting the rotation angle of the output polarization state. Theoretically, the device might find potential applications in variable media excitation, terahertz communication, and terahertz radar ranging.

## Graphical abstract

**Figure:**
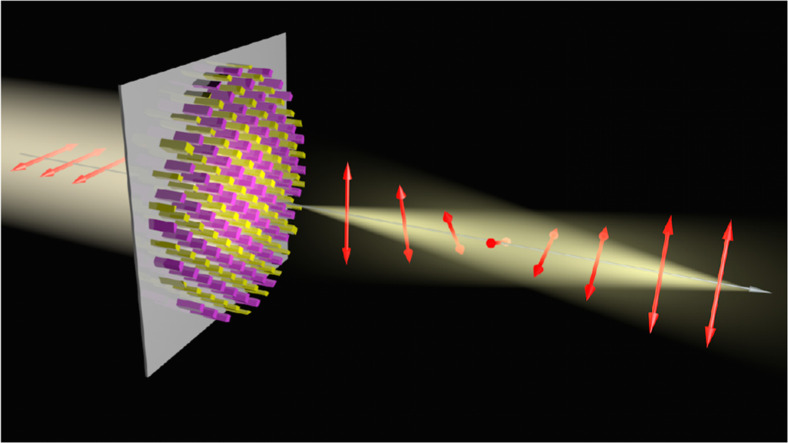

The longitudinal THz wave polarization manipulation: when a THz linearly polarized wave irradiates the device, the transmitted wave forms Bessel beam and obtains polarization states with different rotation angles along the propagation path.

## 1 Introduction

The metasurface platform possesses a remarkable capacity to manipulate the polarization state of electromagnetic waves and can be utilized in various fields such as polarization imaging, polarization detection, space communication, and advanced manufacturing [1], [2], [3], [4], [5], [6]. Metasurfaces are not only capable of imitating traditional optical elements to achieve polarization transformation, but can also integrate polarization with nearly all inherent properties of electromagnetic waves, including phase, frequency, amplitude, orbital angular momentum, and spin angular momentum. This allows for extensive control over the polarization state [7], [8], [9], [10], [11], [12], [13], [14], [15], [16], [17], [18] and promotes advanced physics research [19], [20]. Unfortunately, conventional electromagnetic metasurfaces possess a shared limitation: the design of the Jones transfer matrix focuses on the amplitude and phase changes of waves with diverse polarization components in the *x-y* plane, failing to take into account spatial propagation distance. Therefore, the phase delay of transmitted waves with the increase of propagation distance is the mean variation. As a result, conventional electromagnetic metasurfaces are restricted to manipulating polarization in the two-dimensional space of the *x-y* plane, signifying that the polarization remains constant in each output plane along the propagation path [21], [22], [23]. But how can we develop the ability to manipulate electromagnetic polarization in three-dimensional space to change the polarization state of an electromagnetic wave in various output planes along the propagation path?

To answer the question above, it is necessary to consider the factors of space propagation distance. A metasurface device composed of a series of polarization conversion unit cells with a given phase arrangement can effectively decompose the polarization state of a uniformly incident scalar light field, and modulate the spatio-temporal characteristics of different polarization components to manipulate their phase difference along the spatial propagation path. Once these light components are recombined, the polarization state of incident light can be modified on different output planes. For example, the linearly polarized visible light rotates along the propagation path [24]. The idea of polarization decomposition, independent modulation, and recombination of the incident light field can also be realized by spin-decoupling technology, to obtain the longitudinal control of the light field [25], [26], [27]. The spin-decoupling-based longitudinal polarization control technology has achieved the conversion of a linearly polarized visible light into a three-dimensional vector light field [25]. In the terahertz (THz) band, the spin-decoupling combined with multifocal phase design or focal-depth design enables the output of varying vortex vector THz fields along the propagation path [26,27]. However, the longitudinal THz polarization manipulation proposed in previous reports is limited to several isolated locations [26] or within a short distance (< 2 mm) along the propagation path [27]. The continuous longitudinal manipulation of the polarization state of THz waves over an extended propagation distance has not been achieved. Therefore, new technical routes need to be explored to provide abundant candidate schemes for 3D THz field manipulation and application.

Our work is to realize a metasurface for THz polarization control that utilizes an innovative technical approach, which can achieve user-defined polarization changes on incident light by projecting a longitudinal variable function onto different output planes. In this study, we have achieved the longitudinal continuous manipulation of the polarization state of THz waves within a propagation distance of more than 1 cm, which is a substantial advancement compared to the previously reported manipulation distance of less than 2 mm [27]. Our designed metasurface can decompose the incident linearly polarized THz waves into two orthogonal circularly polarized THz waves with different phase delays. These two circularly polarized components are further recombined to form a linearly polarized THz wave within a certain region (> 1 cm) on the *z*-axis. The phase difference between the two circularly polarized components varies with the propagation distance, causing the combined linearly polarized THz electric field to continuously rotate enough to cover the equator on the Poincare ball. This new THz metasurface device has some potential applications such as variable media excitation, THz communication, THz radar ranging, and refractive index sensing.

## 2 Conceptual design and physical realization of devices

### 2.1 Conceptual design

In general, any in-plane polarized light can be represented by a 2 × 1 Jones matrix, and this representation is generally equal for all output planes. To obtain an element with a linearly polarized state that changes with propagation distance, it is necessary to modify the values of the two entries in the 2 × 1 Jones matrix for each output plane. To achieve this goal, we will redescribe the recombination and decomposition processes between linearly polarized and circularly polarized waves. Suppose that the *x*-polarized wave incident on metasurface is first decomposed into a left-handed circularly polarized (LCP) wave and a right-handed circularly polarized (RCP) wave, and further reassembled into a linearly polarized wave during propagation. Herein, we define the LCP and RCP waves as the anti-clockwise and clockwise motions of the electric field vector along the light propagation direction, respectively. Assuming that light propagation (along the *z*-axis) is not taken into consideration, it means that LCP and RCP waves will have the same phase delay at every point along the *z*-direction, where the phase angle is expressed as *φ*_z_, the normalized Jones matrix of output wave amplitude can be expressed as:

(1) $$\text{LCP}+\text{RCP}=\frac{1}{2}e^{i\varphi_{z}}\left(\begin{array}{c} 1\\ -i \end{array}\right)+\frac{1}{2}e^{i\varphi_{z}}\left(\begin{array}{c} 1\\ i \end{array}\right)=e^{i\varphi_{z}}\left(\begin{array}{c} 1\\ 0 \end{array}\right)$$

Eq. 1 is the simplest and most well-known form of the composition of two circularly polarized waves into a linearly polarized wave. It indicates that the LCP and RCP waves are combined into an *x*-polarized wave, and the polarization state is the same everywhere along the propagation path, which is a very common case. Furthermore, considering the effect of light propagation, the phase delays of LCP and RCP waves propagating to the *z*-axis are different. Assuming that the two circularly polarized waves at a point *z*_0_ in the *z*-axis exhibit the phase difference of*Δφ*, thus the phase delays are *exp*(*iφ*z_0_) and *exp*(*iφ*z_0_ + *Δφ*), respectively, and Eq. 1 therefore changes into:

(2) $$\begin{array}{ccc} \text{LCP}{|}_{z_{0}}+\text{RCP}{|}_{z_{0}} & = & \frac{1}{2}e^{i(\varphi_{z_{0}}+\Delta \varphi )}\left(\begin{array}{c} 1\\ -i \end{array}\right)+\frac{1}{2}e^{i\varphi_{z_{0}}}\left(\begin{array}{c} 1\\ i \end{array}\right)\\ & = & \frac{1}{2}e^{i\varphi_{z_{0}}}e^{i\Delta \varphi /2}\left[e^{i\Delta \varphi /2}\left(\begin{array}{c} 1\\ -i \end{array}\right)+e^{-i\Delta \varphi /2}\left(\begin{array}{c} 1\\ i \end{array}\right)\right]\\ & = & e^{i\varphi_{z_{0}}}e^{i\Delta \varphi /2}\left(\begin{array}{c} \cos(\Delta \varphi /2)\\ \sin(\Delta \varphi /2) \end{array}\right) \end{array}$$

The equation above represents a linearly polarized wave with an angle of Δ*φ*/2 to the *x*-axis. It can be considered as a result of the *x*-polarized wave rotating by Δ*φ*/2 in the *x-y* plane, indicating that the phase difference applied to the LCP and RCP components can deflect the combined linearly polarized wave.

**Fig. 1:**
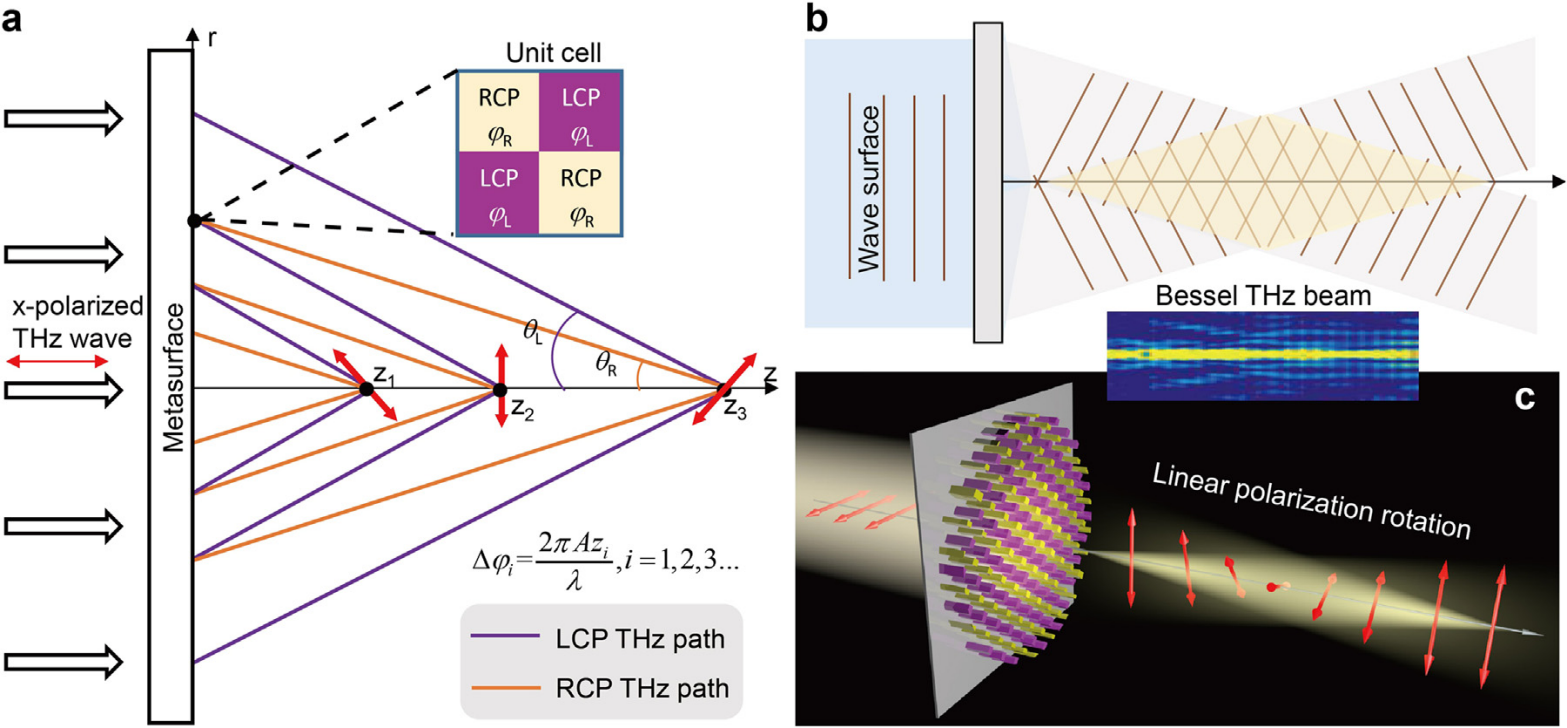
(a) THz optical path diagram based on metasurface. By controlling the phase delays of LCP and RCP waves by unit cells, metasurface can transmit the LCP and RCP components of the incident linearly polarized waves to the z-axis in different paths, and each point in a certain region on the *z*-axis collects the LCP and RCP waves generated by different unit cells respectively. The two kinds of circularly polarized waves are combined again to form linearly polarized waves, and the output linear polarization angle varies with the propagation distance. (b) The phase profile of Eq. 4 makes the output wave form Bessel beam. (c) Functional diagram of a longitudinally polarization variable device, where output linearly polarized THz waves rotate along the propagation path.

Obviously, if Δ*φ* is a fixed value independent of *z*, the element values in the 2 × 1 Jones matrix cannot be modified longitudinally; that is, the polarization state in each output plane remains unchanged. To further change the linear polarization direction in Eq. 2 with the light propagation, Δ*φ* must be a function of *z*, denoted as Δ*φ* = Δ*φ*(*z*), which can be achieved by designing the optical paths of LCP and RCP waves. The optical path design of LCP and RCP waves can be achieved on the all-dielectric metasurface. As illustrated in Fig. 1a, each unit cell can transmit the LCP and RCP components to the *z*-axis with different phase delays, and every single point in a certain region of the *z*-axis can collect the LCP and RCP waves generated by different unit cells (see section S1 in Supplementary Materials), and further two kinds of circularly polarized waves are combined again to form linearly polarized waves. As demonstrated in Fig. 1a, by controlling the phase delays of LCP and RCP waves, a unit cell on the metasurface can transmit the RCP wave (purple line) and LCP wave (brown line) to *z*_2_ and *z*_3_ on the *z*-axis respectively, and the LCP wave from a unit cell and the RCP wave from another unit cell converge to *z*_3_ to form a linearly polarized wave. The LCP wave paths from all unit cells process the same slope with an angle of *θ*_L_ about the *z*-axis, and the RCP wave paths from all unit cells process the same slope with an angle of *θ*_R_ about the *z*-axis, where *θ*_L_≠*θ*_R_. Hence, based on the geometric relationship, it is easy to deduce that the optical paths of LCP and RCP waves transmitted to a point on the *z*-axis are *z*/cos*θ*_L_ and *z*/cos*θ*_R_ respectively, and the phase difference of the two circularly polarized components is:

(3) $$\Delta \varphi \left(z\right)=\varphi_{\text{LCP}}-\varphi_{RCP}=\frac{2\pi Az}{\lambda }$$

where *λ* is the working wavelength, and *A* is a constant expressed as *A* = 1/cos*θ_L_* − 1/cos*θ*_R_. The Δ*φ*(*z*) monotonously changes along the *z*-axis, which leads to the linearly polarized wave expressed in Eq. 2 rotating with the light propagation. The required phase profile for the metasurface meeting Eq. 3 is (see section S1 in Supplementary Materials):

(4) $$\left\{ \begin{array}{c} \phi_{L\text{CP}}=-\frac{2\pi }{\lambda }\left(r\sin\theta_{L}\right)\\ \phi_{R\text{CP}}=-\frac{2\pi }{\lambda }\left(r\sin\theta_{R}\right) \end{array}\right.$$

where *r* is the distance from a point on the metasurface to the metasurface center; since the metasurface is located in the *x-y* plane, we have $r=\sqrt{x^{2}+y^{2}}$. The phase distribution of Eq. 4 enables LCP and RCP wavefronts to propagate as shown in Fig. 1b, further forming a Bessel beam. The function of the device is depicted in Fig. 1c. When a linearly polarized THz wave is incident on the metasurface device, the transmitted wave takes on the form of a Bessel beam and acquires polarization states with varying rotation angles as it propagates, ultimately achieving longitudinal THz wave polarization control.

### 2.2 Device design and experiment

**Fig. 2:**
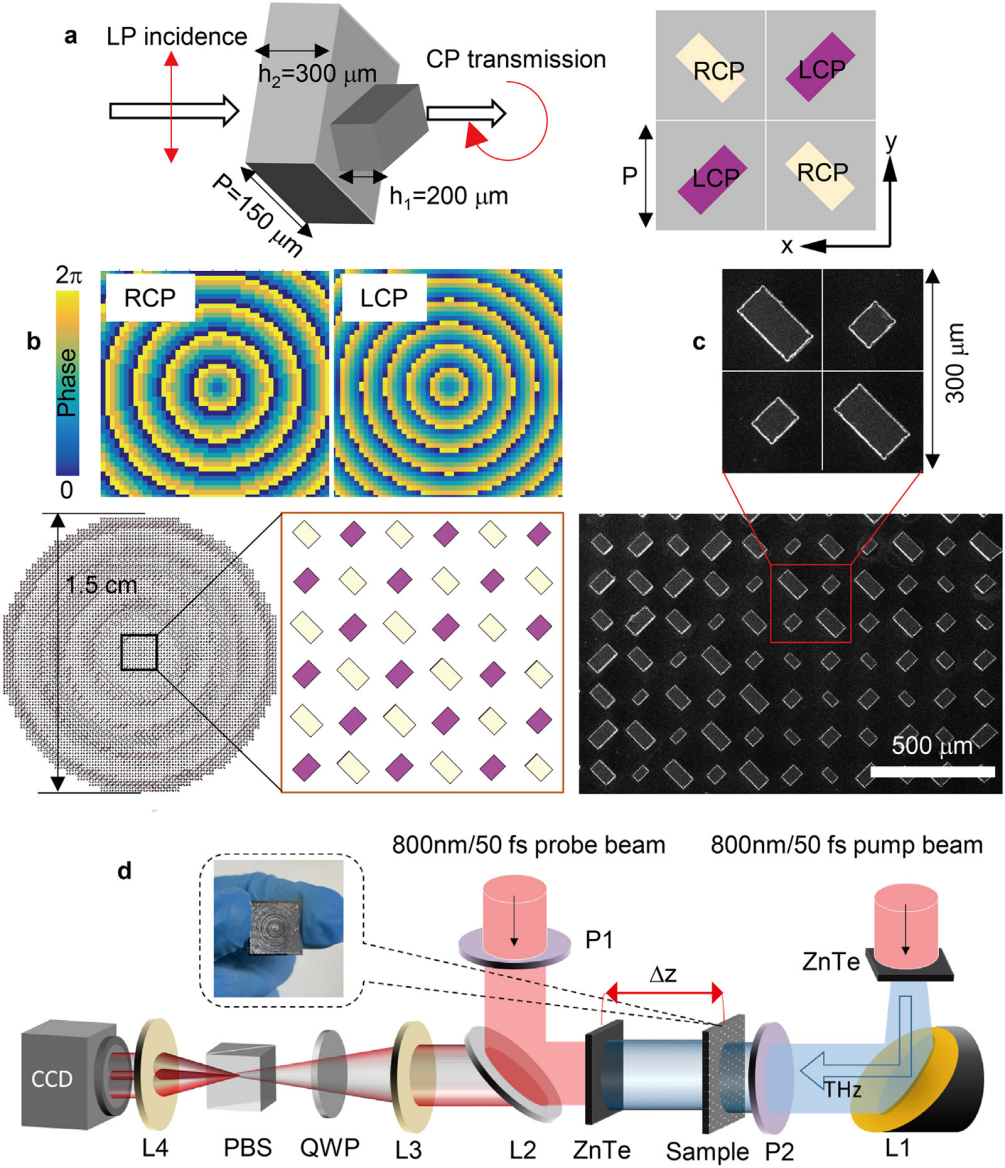
(a) The schematic diagram of sub-units: a complete unit cell is composed of four sub-units, in which two identical purple sub-units control the propagation of LCP component and two identical yellow sub-units control the propagation of RCP component. (b) The required phase profile for RCP and LCP components and the schematic device, corresponding to the SEM images of sample in (c). (d) The schematic diagram for the measured setup. By moving the sample to adjust the distance Δ*z* between the sample and the ZnTe detecting crystal, the amplitude and phase information of THz wave at different output planes can be characterized, and the THz polarization information can be further obtained.

We used CST microwave studio software with the time-domain solver to simulate the metasurface, where the boundary conditions of the unit cell are ‘period’ in the *x-y* direction and ‘open’ in the *z*-direction, the THz plane wave was selected as the excitation source. The metasurface requires each unit cell can synchronously manipulate the LCP and RCP components of the incident linearly polarized THz wave. As illustrated in Fig. 2a, a silicon (the relative permittivity *ε* = 11.9 and resistivity *ρ* > 10,000 Ω⋅cm) rectangular bar with 45° inclination on the *x-y* plane can obtain the co-polarized and cross-polarized transmission waves with the same amplitude, and the phase difference between the two transmission waves remains a constant |π/2|, which is similar to a quarter wave plate and ultimately outputs a circularly polarized wave (see section S2 in Supplementary Materials). According to the phase characteristic of Pancharatnam-Berry (PB), a silicon rectangular bar can be rotated to 90° to obtain circular polarization output with an opposite spiral direction. As illustrated in Fig. 1a (right), a rectangular bar (purple marked) outputting RCP THz wave, after being rotated with 90°, outputs LCP THz wave (yellow mark). A complete unit cell consists of four sub-units that perform linear-circular polarization conversion. Within the unit cell, two identical sub-units control the RCP component, and the other two control the LCP component and are arranged in a spatial interleaving manner. Thus, the transmission matrix of the whole unit cell is described as the superposition of the two transmission matrices (see section S2 in Supplementary Materials):

(5) $$T\left(z\right)=T_{L}\left(z\right)+T_{R}\left(z\right)=\frac{1}{2}Ce^{i\varphi_{L}}\left(\begin{array}{cc} 1 & -i\\ -i & 1 \end{array}\right)+\frac{1}{2}Ce^{i\varphi_{R}}\left(\begin{array}{cc} -1 & -i\\ -i & -1 \end{array}\right)$$

where the first term represents the transfer matrix of the output LCP wave and the second one represents the transfer matrix of the output RCP wave respectively, *C* is a constant.

To control the wavefront of the circularly polarized wave component, six basic structures (see section S2 in Supplementary Materials) are selected. Their relative phase delays cover 0–2π with the spacing of π/3. The phase profile and device schematic diagram required for RCP and LCP components are shown in Fig. 2b. The phase profile is determined by Eq. 4. The designed operating frequency for this work is 1.05 THz, sin*θ_L_* = 0.38 and sin*θ_R_* = 0.27, and the entire device diameter is 1.5 cm. The device material uses 500 μm thick high resistivity all-silicon (*ε* = 11.9 and *ρ* > 10,000 Ω⋅cm). The device is fabricated with Inductively Coupled Plasma (ICP) etching technology as shown in Fig. 2c. The measured setup is shown in Fig. 2d [28], a femtosecond laser beam with a wavelength of 800 nm excites ZnTe nonlinear crystal to generate THz beam. The THz beam is collimated by the ellipsoidal reflector L1, and the polarization state is defined by the polarizer P2, ultimately incident on the metasurface sample. The transmitted THz beam is received by the second ZnTe crystal. The detection part uses electro-optical sampling technology, where the 800 nm femtosecond laser detection beam with polarization state is controlled by the polarizer P1, passes through the semi-transparent mirror, further excites the ZnTe crystal at the transmission side, and finally reflects back a THz-modulated 800 nm laser. The reflected laser carries the THz wave information and passes through the focusing lens L3, the quarter wave plate (QWP), the Wollaston prism and the focusing lens L4 in turn, further received by the CCD camera to complete the electro-optical sampling process, and finally the transmitted THz wave information of the metasurface sample is analyzed. By moving the sample, changing the distance between sample and ZnTe detecting crystal, Δ*z* can gain THz wave information at different output planes.

## 3 Results and discussion

**Fig. 3:**
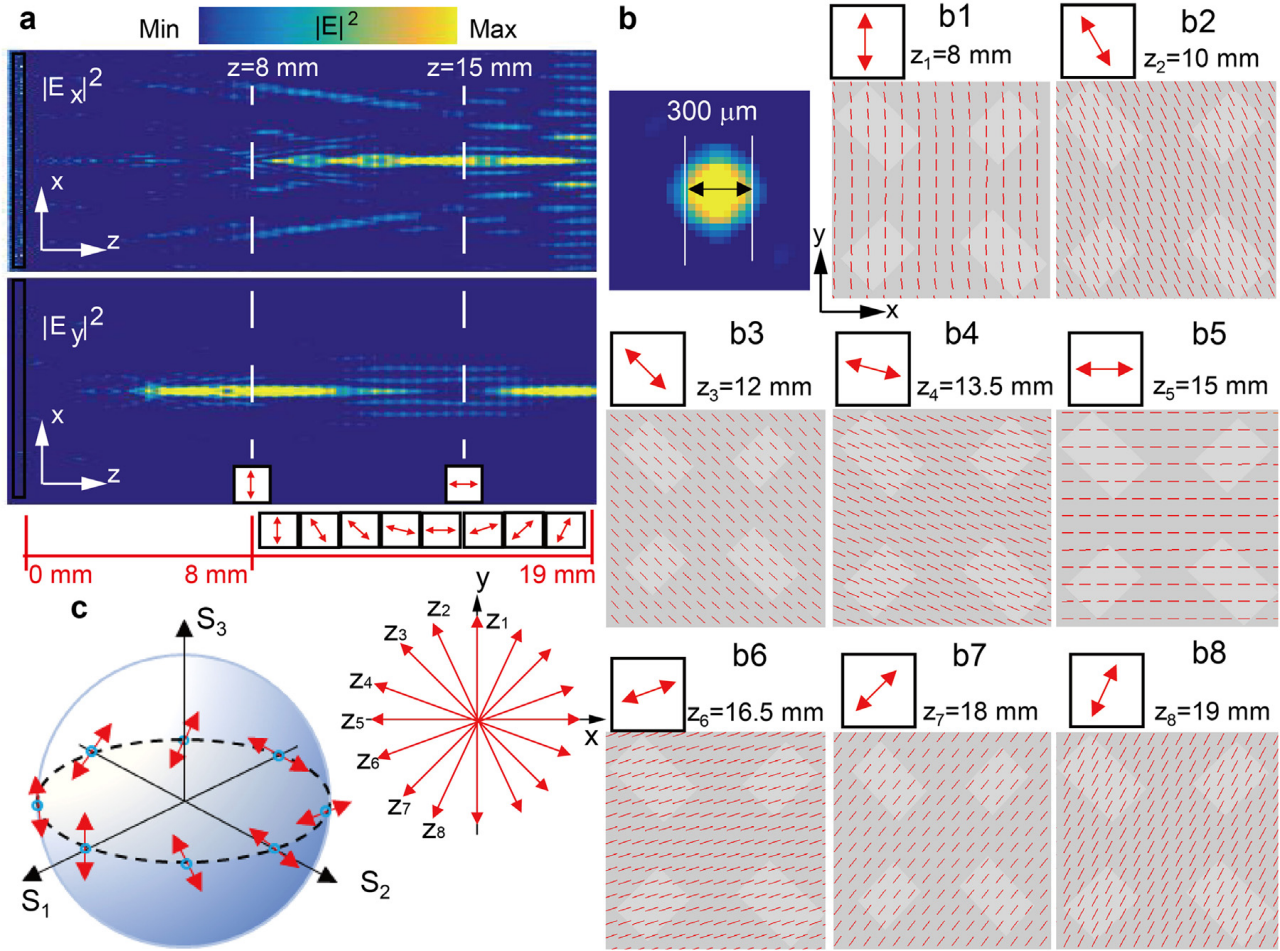
(a) The simulative electric field intensity distribution for the *x*-polarized and *y*-polarized components of the output wave in the *x*-*z* plane. (b) The simulation results of electric field intensity distribution (|E*_x_*|^2^ + |E*_y_*|^2^) of output wave in the *x-y* plane, and the THz spot diameter is about 300–400 μm; (b1-b8) show the simulative polarization states of the THz spots at different positions along the propagation path, where the THz propagation direction vertically points out of the paper. (c) The polarization state distribution corresponding to (b1-b8) on Poincare sphere (left) and their two-dimensional view in the *x-y* plane (right).

To determine the rotational change of the transmitted linear polarization state in the *z* direction, we first focused on the intensity changes of the *x*-polarized and *y*-polarized components in the *x-z* plane, as demonstrated in Fig. 3a. As it depicts in Fig. 3d, the longitudinal monitoring ranges from *z* = 8 mm to *z* = 19 mm (the distance over 1 cm) with the monitoring frequency at 1.05 THz, and the beam diameter is 300–400 μm which is close to the size of a complete unit cell. With the transmission polarization rotation, the intensity distributions of the *x*-polarized and *y*-polarized components change inevitably. As shown in Fig. 3a, the polarization states of two points can be easily determined, which are located at *z* = 8 mm and *z* = 15 mm, respectively. At *z* = 8 mm, the electric field shows the intensity of the *y*-polarized component, but none of the *x*-polarized component, which indicates a *y*-polarized output wave; at *z* = 15 mm, the electric field intensity of the *y*-polarized component is zero and the *y*-polarized component exists, which indicates an *x*-polarized output wave. From *z* = 8 mm to *z* = 15 mm, the output wave goes through the evolution from the *y* polarization state to the *x* polarization state. The change rules of the output polarization state can be further understood by detecting the distribution of electric field lines in the beam center on different planes. The simulation results are shown in Fig. 3b1-b8. When the polarization states are projected onto an *x-y* plane, it is easy to find the polarization states rotate counterclockwise along the propagation path, as shown in Fig. 3c (right). In detail, the output waves with a propagation distance between *z* = 8 mm and *z* = 15 mm experience the rotation from the *y* polarization state to the *x* polarization state (*z*_1_-*z*_5_), and the electric field oscillates through the second and fourth quadrants. Subsequently, the output waves with a propagation distance between *z* = 15 mm and *z* = 19 mm rotate from the *x* polarization state to the *y* polarization state (*z*_5_-*z*_8_), and the electric field oscillates through the first and third quadrants. Furthermore, the polarization states of eight points (*z*_1_-*z*_8_) are mapped onto the Poincare sphere, as shown in Fig. 3c (left). These output polarization states appropriately cover the equator of the Poincare sphere.

**Fig. 4:**
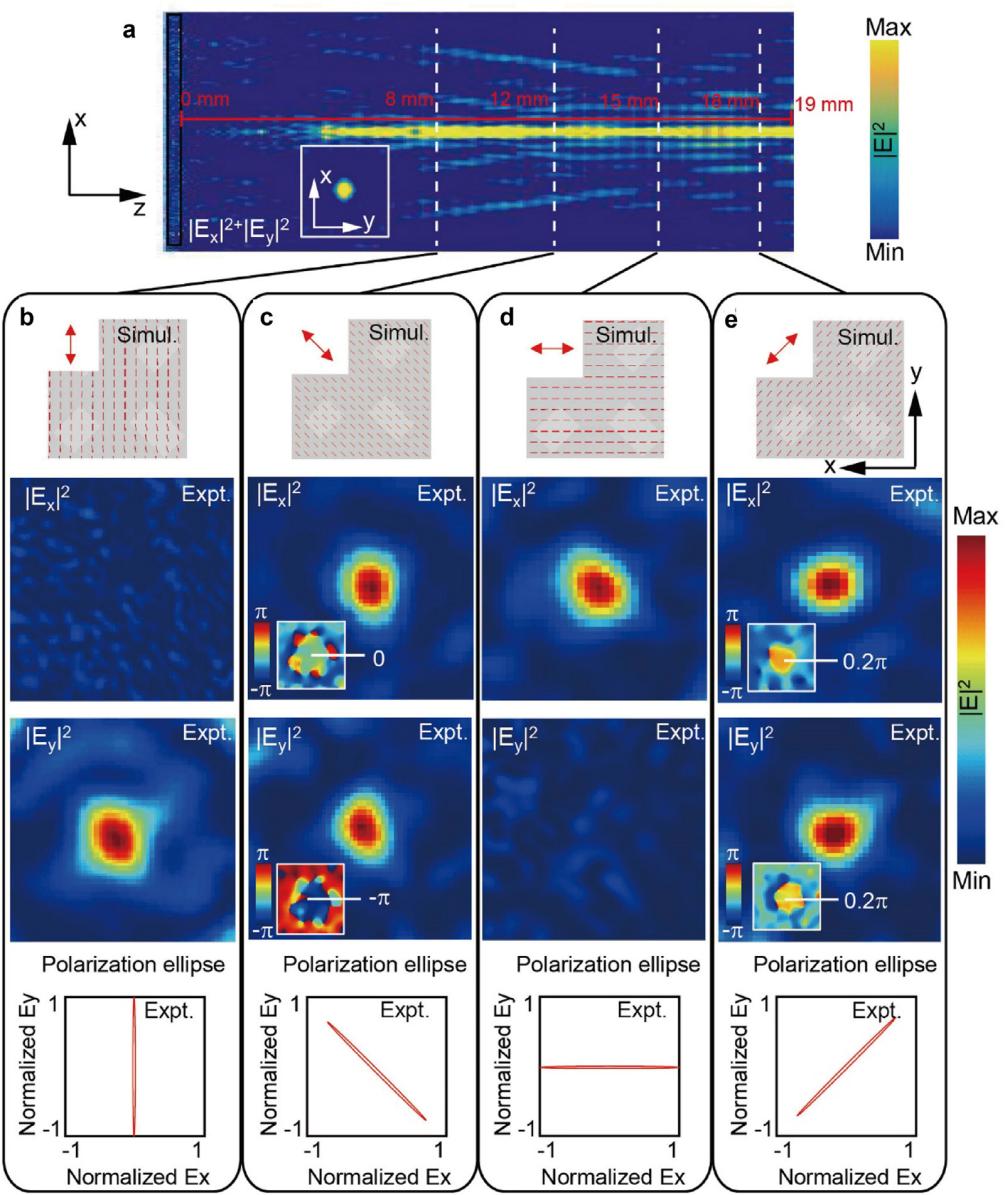
(a) The total electric field intensity distribution of output wave in the *x-z* plane. (b-e) The simulated polarization state distribution at light spot, corresponding to experimental results for electric field intensity and phase distribution of the *x*- and *y*- polarized components, as well as polarization ellipse, at four points on the propagation path, including *z* = 8 mm (b), *z* = 12 mm (c), *z* = 15 mm (d) and *z* = 19 mm (e). The propagation direction vertically points out of the paper, and the polarization ellipses are plotted via the amplitude and phase of the *x*- and *y*- polarized components at the center of light spot.

By observing the total intensity |E*_x_*|^2^ + |E*_y_*|^2^ on the *x-z* plane, a complete Bessel beam can is observed as shown in Fig. 4a. To confirm the simulation results, we tested the amplitude and phase distributions of electric field components to depict polarization state at several key points, as shown in Fig. 4b-e. At *z* = 8 mm, the simulation results show a *y*-polarized THz output wave, corresponding to the experimental results that only show the electric field intensity of the *y*-polarized component with no *x*-polarized component (Fig. 4b). As the THz wave propagates to *z* = 12 mm, the simulation results show a 45° inclined linear polarization field distributed in the second and fourth quadrants; the measured results indicate that the electric field intensities of the *x*-polarized and *y*-polarized components are generally the same, and the phase of *y*-polarized electric field lags behind that of the *x*-polarized electric field by π (Fig. 4c), indicating a linear polarization field (with very weak detected ellipticity) distributed in the second and fourth quadrants inclined at 45°, which is consistent with the simulation results. At *z* = 15 mm, the output THz polarization state rotates into the *x*-axis, therefore only the THz electric field is detected in the *x*-polarized component, and the experimental results correspond well with our simulation results (Fig. 4d). Further, when *z* = 18 mm, the simulation results indicate that the *x*-polarized THz rotates counterclockwise into the first and third quadrants to form a 45° inclined linearly polarized wave, which can also be proved by the experimental results (with very weak detected ellipticity): the electric field intensities of the tested *x*-polarized and *y*-polarized components are generally the same, and the phase difference between them is almost zero (Fig. 4e).

The longitudinal polarization variable THz meta-device provides users with more polarization customization solutions and can benefit some applications. For instance, some electromagnetic response mediums (i.e., electro-optical crystals) are sensitive to the intensity and polarization of THz waves; the metasurface can spatially adjust the THz waves to modify the excited intensity and excited mode of the medium, therefore obtaining different output information. Meanwhile, in THz high-speed communication, the longitudinal variable polarization makes the communication more confidential for the polarization information intercepted at different positions on the propagation path is different, which brings some difficulties in deciphering information. Additionally, the terminal receives different polarization states through a moving device, which can be used as an information transmission mode. As the polarization transform is a function of the propagation distance, it is a promising candidate for THz radar: the THz radar can identify the movement of the target by emitting THz waves with the longitudinal variable polarization to the target and detecting the polarization change of reflected THz waves. Moreover, the propagation of the THz wave is related to the refractive index of the propagation medium; therefore placing additional media on the propagation path can change the polarization state of the output wave, which can be used as a new sensing scheme to detect the unknown refractive index.

**Fig. 5:**
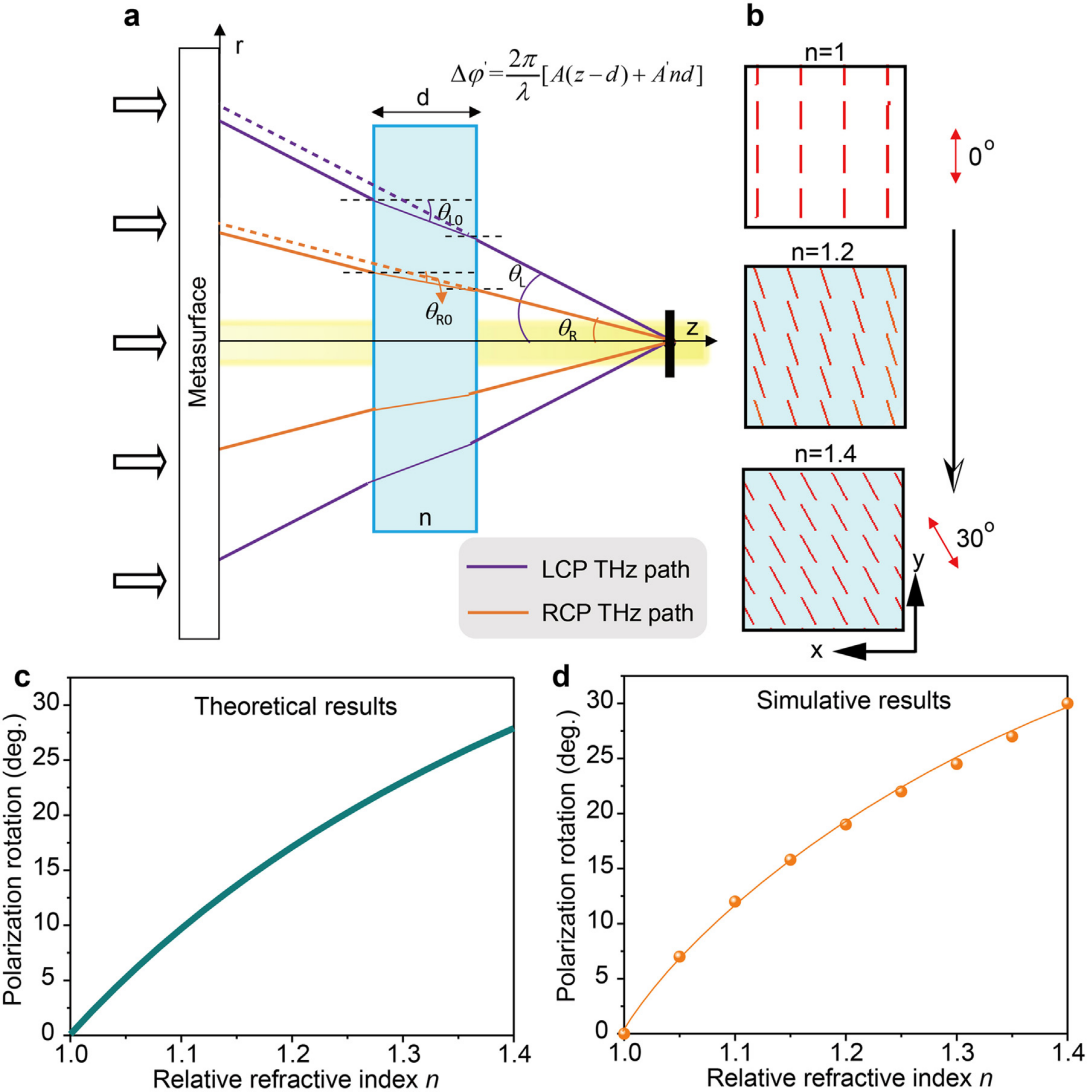
(a) The schematic concept for the refractive index sensing. Placing additional medium in the propagation path will change the phase difference between LCP and RCP components. The refractive index is determined by detecting the change of polarization state in an output plane. (b) With the change of refractive index, the viewed polarization state in the *z* = 8 mm plane rotates, where the propagation direction vertically points out of the paper. (c-d) Theoretical and simulation results of *P*_rot_ vs. *n*.

Here, we take the refractive index sensing as a demonstration of the application concept. Fig. 5a demonstrates the schematic diagram of the proposed refractive index sensing application. A loss-free medium with a thickness of *d* and a relative refractive index of *n* (cyan rectangular block in Fig. 5a) is placed on the THz propagation path. The reflection caused by the detected medium is relatively weak (see section S3 in Supplementary Materials), which avoids the multiple interferences of THz wave between the medium and the metasurface, and ensures the accuracy of the simulation results as much as possible. Because the refractive index of the medium is different from that of the vacuum, it will inevitably affect the phase difference between LCP and RCP waves, resulting in the change of polarization state at a fixed point on the *z*-axis. When an additional medium is inserted into the propagation path, the phase difference between LCP and RCP waves is not only related to *z*, but also related to the thickness and refractive index of the medium. By analyzing the geometric relationship in Fig. 5a, it is easy to calculate the phase difference between LCP and RCP waves converging to a point on the *z*-axis using the following equations:

(6) $$\begin{array}{ccc} & & \Delta \varphi^{\prime }\left(z,d,n\right)=\frac{2\pi }{\lambda }\left[A\left(z-d\right)+A^{\prime }nd\right]\\ & & A^{\prime }=\frac{1}{\cos\theta_{L0}}-\frac{1}{\cos\theta_{R0}}=n\left(\frac{1}{\sqrt{n^{2}-\sin^{2}\theta_{L}}}-\frac{1}{\sqrt{n^{2}-\sin^{2}\theta_{R}}}\right) \end{array}$$

where the medium with the thickness of *d* = 3 mm is set. We detect the transmission polarization state in the *z* = 8 mm plane under the *x*-polarized THz wave incidence. As discussed above, in vacuum, i.e. *n* = 1, the output THz wave at *z* = 8 mm is a *y*-polarized state. Nevertheless, with the addition of medium where the refractive index increases to *n* = 1.4, the simulation results indicate that the polarization state of the output THz wave in the *z* = 8 mm plane rotates counterclockwise by 30°, as shown in Fig. 5b. Herein, the angle of the output polarization state relates to that at *n* = 1 is defined as the polarization rotation angle, expressed as *P*_rot_. Theoretically, *P*_rot_ can be described as follows:

(7) $$\begin{array}{ccc} & & P_{rot}=\pi -\Delta \varphi^{\prime }/2-P_{1}\\ & & P_{1}=\pi -\Delta \varphi^{\prime }{|}_{n=1}/2 \end{array}$$

where *z* = 8 mm and *d* = 3 mm have been defined, thus the true independent variable in Δ*φ*′ is only *n*. It can be deduced from Eq. 2 that the angle of the original output polarization state concerning the *x*-axis is half of the phase difference between the LCP and RCP waves, i.e. Δ*φ*′/2, with the precondition that the polarization state is observed along the propagation direction. On the contrary, the polarization state is observed toward to propagation direction here, therefore the coordinate transformation should be taken into consideration. The sight direction for monitoring the polarization state is opposite to the THz propagation direction, causing the *x-y* plane to flip forward and backward; that is, the coordinate transformation is (x,y)→(−x,y). Therefore, the viewed angle of the polarization state for the *x*-axis should be π−Δ*φ*′/2; when *n* = 1, the angle of the polarization state is *P*_1_. In addition, since we only consider the rotation angle of the output polarization state related to that at *n* = 1, *P*_rot_ is finally expressed in Eq. 7. The theoretical trend described based on Eq. 7 is shown in Fig. 5c. The relationship between *P*_rot_ and *n* is further simulated and depicted in Fig. 5d, which is generally consistent with the theoretical trend in Fig. 5c. It should be noted that the phase profile of metasurface is composed of discrete phases rather than ideal continuous phases, and the theoretical and simulation results exhibit slight difference. The sensitivity of refractive index sensing calculated based on the simulation results is *S* = 75°/RIU, where *S* is defined as *S* = *P*_rot_/Δ*n*, indicating the rotation angle change of output polarization state is caused by refractive index unit (RIU) change. It should be noted that the relationship between *P*_rot_ and *n* is not linear, which can also be seen from the derivation of *P*_rot_ on *n*. Obviously, ∂*P*_rot_/∂*n* is not a constant, but a variable related to *n*.

## 4 Conclusion

In summary, we successfully realized a THz polarization manipulation along the propagation path based on an all-dielectric metasurface. The device enables the incoming *x*-polarized THz wave to transform into a transmitted linearly polarized wave that rotates with the propagation distance by an all-silicon metasurface. Each unit cell of the metasurface is interlaced by four sub-units that control the transmission of LCP and RCP waves and can decompose the incoming *x*-polarized wave into LCP and RCP waves, and impose different phase delays on the two components. All the components can converge on the *z*-axis to form Bessel beams. The linearly polarized wave is obtained within a relatively long distance on the *z*-axis which is composed of LCP and RCP components with different phases generated by different unit cells. The phase difference between the LCP and RCP components at a point on the *z*-axis is designed to vary monotonically with propagation distance at a point on the *z*-axis. As a result, the combined linearly polarized state undergoes continuous rotation along the propagation path, with the rotation angle ranging from 0 to π, covering the equator of the Poincare sphere. Finally, based on the longitudinal polarization variable property, we demonstrate a new refractive index sensing application. An additional medium is introduced in the propagation path. With the change in the refractive index of the medium, the polarization in an output plane rotates, and the unknown refractive index is detected.

## Author contributions

JT Li conceived the idea, designed the experiments and wrote original manuscript. J Liu performed the key measurements, analyzed the experimental data. Z Yue, J Li and C Zheng performed a part of experiments. F Yang and H Li contributed to the simulation and drew the graphics. YT Zhang, Y Zhang and J Yao reviewed manuscript and supervised progress.

## Declaration of competing interest

The authors declare that they have no conflicts of interest in this work.

## Appendix Supplementary materials

Image, application 1
