# Supplementary material for: Polarization variable terahertz metasurface along the propagation path
Source: Fundam Res. 2023 Apr 28;5(1):124–31. doi: 10.1016/j.fmre.2023.03.017 (PMC11955037; doi:10.1016/j.fmre.2023.03.017)
Supplement: Supplementary file 1 [file mmc1.docx]

**<Supplementary Materials>**

**Polarization variable terahertz metasurface along the propagation path**

Jitao Li^1^*, Jingyu Liu^2^, Zhen Yue^1^, Jie Li^1^, Chenglong Zheng^1^, Fan Yang^1^, Hui Li^1^, Yating Zhang^1^*, Yan Zhang^2^*, Jianquan Yao^1^*.

^1^*Key Laboratory of Opto-Electronics Information Technology (Tianjin University), Ministry of Education, School of Precision Instruments and Opto-Electronics Engineering, Tianjin University, Tianjin, 300072, China*

^2^*Beijing Key Laboratory for Metamaterials and Devices, Key Laboratory of Terahertz Optoelectronics, Ministry of Education, and Beijing Advanced Innovation Center for Imaging Technology, Department of Physics, Capital Normal University, Beijing 100048, China*

*Corresponding authors. E-mails: [jtlee@tju.edu.cn](mailto:jtlee@tju.edu.cn) (JT Li); [yating@tju.edu.cn](mailto:yating@tju.edu.cn) (YT. Zhang); [yzhang@cnu.edu.cn](mailto:yzhang@cnu.edu.cn) (Y. Zhang); [jqyao@tju.edu.cn](mailto:jqyao@tju.edu.cn) (J. Yao)

**Contents**

**S1. The phase profile design of metasurface**

**S2. The unit cell and sub-unit design of metasurface**

**S3. The transmittance and reflectivity spectra of the detected medium**

**S1. The phase profile design of metasurface**

By separating the RCP wave transmission path and the LCP wave transmission path, the geometric relationship can be clearly seen, as shown in the upper part of Fig. S1. It is easy to calculate the optical path for LCP wave and RCP wave to the equiphase surface as follows:

$\left\{\begin{matrix} OP_{\mathrm{L}}=r\sin\theta_{\mathrm{L}} \\ OP_{\mathrm{R}}=r\sin\theta_{\mathrm{R}} \end{matrix}\right.$ (S1)

Where *r* is the distance from a point on the metasurface to the metasurface center; since the metasurface is located in the *x-y* plane, we have $\text{r}=\sqrt{x^{2}+y^{2}}$. The included angle between LCP wave path and z-axis is *θ*_L_, and the included angle between RCP wave path and z-axis is *θ*_R_, where *θ*_L_=*θ*_R_. Therefore, in order to obtain the optical path in Eq. (S1), the phase profile required for the metasurface is:

(S2)

Where *λ* is working wavelength. To facilitate understanding, the propagation of LCP wave and RCP wave can be described in the form of beam instead of light line, corresponding to the lower part of Fig. S1. It can be seen that after the LCP and RCP waves are transmitted to the z-axis, they overlap in a region on the z-axis, and they are combined into linear polarization again. In the linear polarization region, LCP and RCP components at each point are not from one unit cell, but from different unit cells. Obviously, LCP and RCP components of each point carry different phase delays, and their phase difference meets the Eq. (3) in main text.


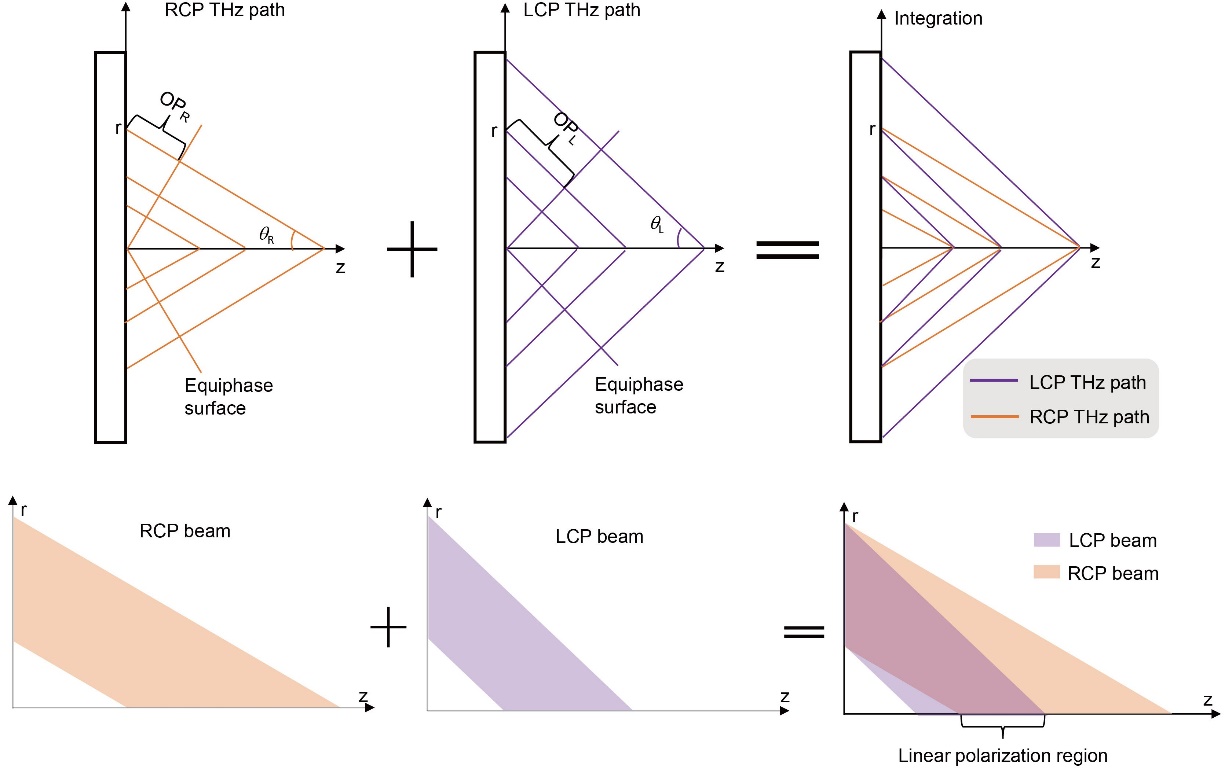


Fig. S1. The schematic diagram of separation and recombination of RCP wave path and LCP wave path (upper part) and corresponding to beam representation (lower part).

**S2. The unit cell and sub-unit design of metasurface**

The THz line-circular polarization conversion can be realized by a simple birefringent silicon rectangular bar. When the *x*- and *y*- polarized waves pass through a silicon rectangular bar parallel or perpendicular to the *x*-axis, there is no cross polarization component in the transmission wave. The transmission Jones matrix ***T*** can be expressed as follows:

(S3)

Where *t_xy_* represents the transmission coefficient of *x*-polarized wave under the incidence of *y*-polarized wave. When the silicon rectangular bar rotates at a certain angle *Φ*, the matrix ***T*** will be changed into a new matrix ***T*_0_** under the coordinate transformation matrix $\text{Λ}_{1}=\left(\begin{matrix} \cos\text{Φ} & -\sin\text{Φ} \\ \sin\text{Φ} & \cos\text{Φ} \end{matrix}\right)$:

(S4)

The electric vector of the incident *x*-polarized wave can be expressed as (1 0)^T^, and the transmission field is:

(S5)

Let *Φ*=π/4, i.e., cos^2^*Φ*=sin^2^*Φ*=1/2, and set |*t_xx_*|=|*t_yy_*|=*t*, where *t* is the transmission coefficient of silicon, which is determined by the physical properties of silicon material. If the reflection and absorption of silicon wafer are ignored, we have *t*=1. Also, let *ϕ_yy_*−*ϕ_xx_*=2*θ* (the value range of 2*θ* is limited to 0-2π, so that the range of *θ* is 0-π), then the above equation is simplified as:

(S6)

Further, let *ϕ_xx_*=*ϕ*−*θ*, and the above equation can be changed into:

(S7)

It is easy to find that when *θ*=π/4 and *θ*=3π/4, the above equation represents a LCP wave and a RCP wave respectively, and a simplified form of the transfer matrix generating LCP wave and RCP wave is obtained simultaneously:

(S8)

Where $C\text{=}\sqrt{2}/2$.

The above deduction shows that the *x*-polarized wave can be converted into LCP wave theoretically after passing through the rectangular bar with π/4 rotation, as shown in Fig. S2a (light incident from the bottom of the structure). In order to control the wavefront, six structures are designed, and the structural parameters are shown in Table S1. The six silicon rectangular bars with π/4 rotation in the *x-y* plane can achieve the *x*- and *y*- polarized transmission waves with the same amplitude, phase coverage of 0-2π, and the phase of the *x*-polarized component lagging behind the *y*-polarized component by π/2, which leads to a LCP wave finally output. The RCP wave can be obtained by further rotating these structures with π/2. The complete unit cell designed in this work is composed of four sub-units that preform linear-circular polarization conversion. The two identical sub-units that control RCP components and the two identical sub-units that control LCP components are arranged in a spatial interleaving manner, as shown on the right of Fig. 2a in main text. Thus, the whole unit cells’ transmission matrix is described as the superposition of two transmission matrices:

(S9)


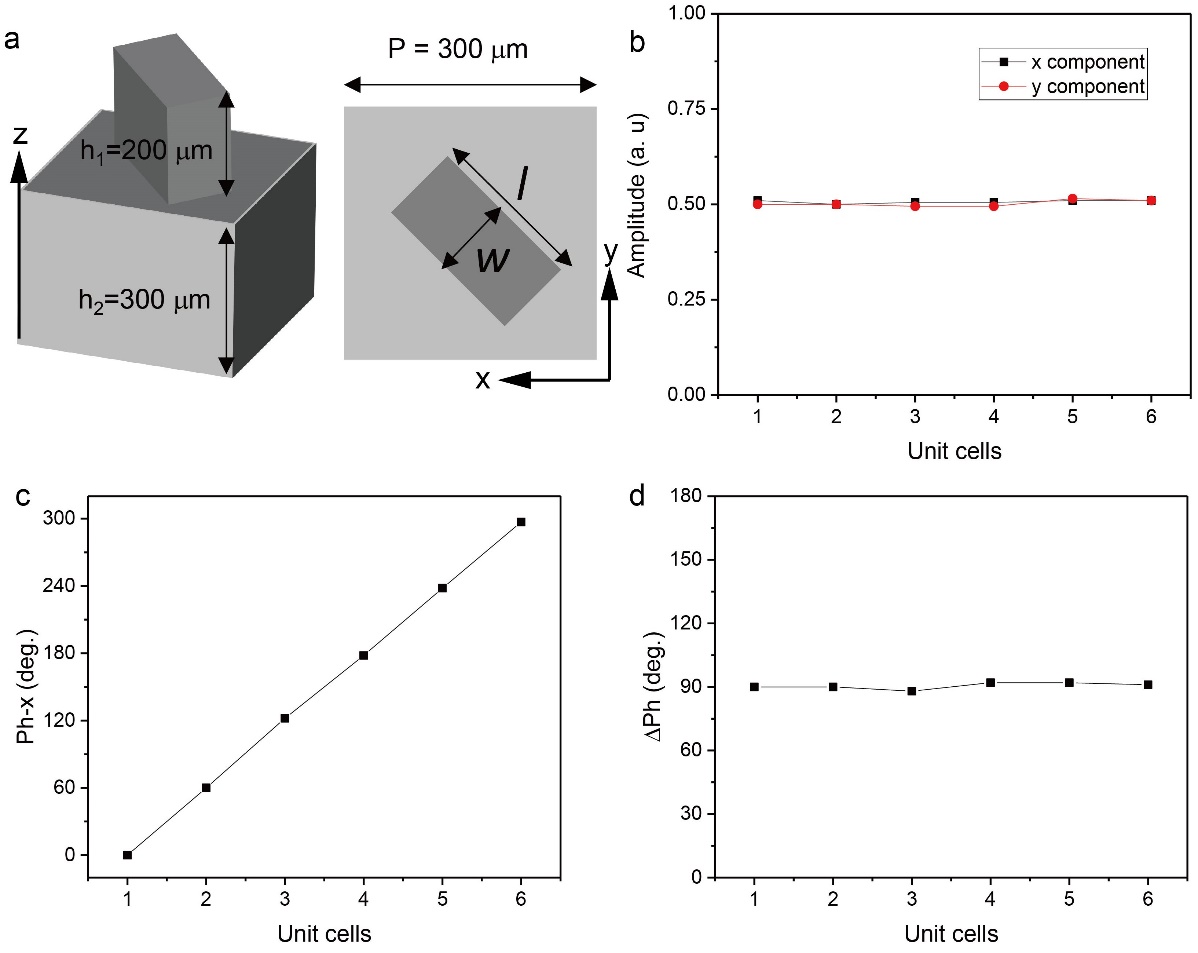


Fig. S2. (a) Side view and top view of sub-unit for linear-circular polarization conversion; six structures are selected, and the parameters are shown in Table S1. When the *x*-polarized wave at 1.05 THz enters these sub-units from the bottom, the *x*- and *y*- polarized component amplitudes of the output wave are shown as (b), the relative phase of the corresponding *x*-polarized component is (c), and the phase difference between the *y*- and *x*- polarized components is (d), where Δ*Ph*=*Phy*−*Phx*.

Table S1. The structural parameters for six sub-units

| Sub unit cells | 1^st^ | 2^nd^ | 3^rd^ | 4^th^ | 5^th^ | 6^th^ |
| --- | --- | --- | --- | --- | --- | --- |
| *w* (μm) | 36 | 48 | 52 | 54 | 56 | 60 |
| *l* (μm) | 62 | 62 | 64 | 72 | 92 | 114 |

**S3. The transmittance and reflectivity spectra of the detected medium**

Under different incident angles, the transmittance and reflectivity of the detected medium are investigated, as shown in Fig. S3. Since we design sin*θ*_L_=0.38 and sin*θ*_R_=0.27, the angle range of 0-60^o^ is enough. The designed medium shows very weak reflectivity, which avoids the multiple interference of THz wave between the medium and the metasurface, and ensures the accuracy of the simulation results as much as possible.


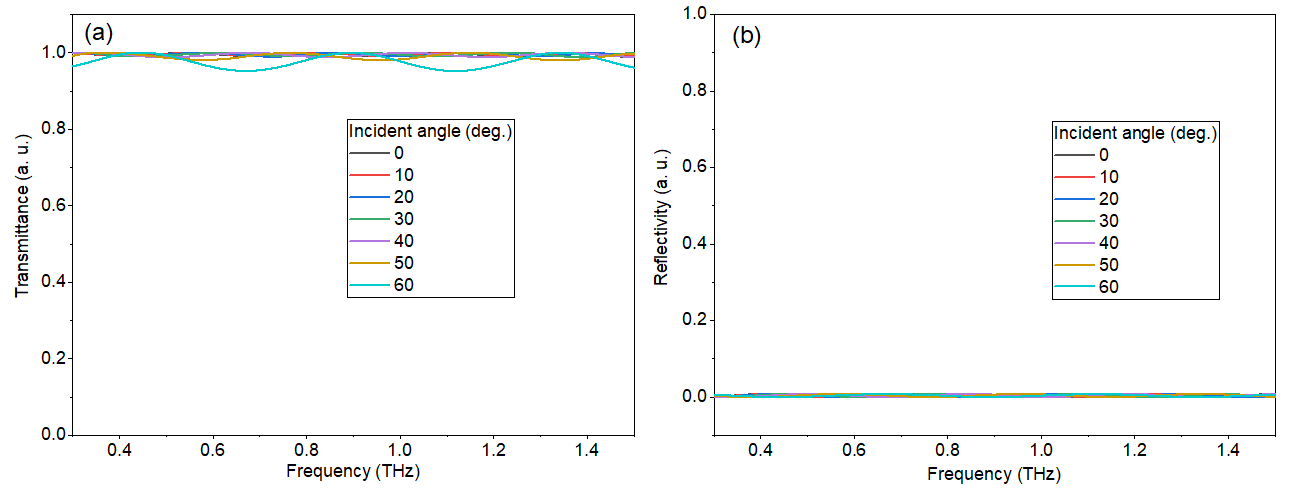


Fig. S3. (a) The transmittance and (b) reflectivity spectra of the detected medium with different incidence angles.
